# Parent & Child Perceptions of Child Health after Sibling Death

**DOI:** 10.15344/2394-4978/2016/185

**Published:** 2016-06-07

**Authors:** Rosa M. Roche, Dorothy Brooten, JoAnne M. Youngblut

**Affiliations:** 1 Cystic Fibrosis/Asthma Center Coordinator, Nicklaus Children’s Hospital, Miami, Florida, USA; 2 Nicole Wertheim College of Nursing & Health Sciences, Florida International University Miami, Florida, USA

**Keywords:** Child Death, Child-rated Health, Infant Death, Parent-rated Child Health

## Abstract

**Background:**

Understanding children’s health after a sibling’s death and what factors may affect it is important for treatment and clinical care. This study compared children’s and their parents’ perceptions of children’s health and identified relationships of children’s age, gender, race/ethnicity, anxiety, and depression and sibling’s cause of death to these perceptions at 2 and 4 months after sibling death.

**Methods:**

64 children and 48 parents rated the child’s health “now” and “now vs before” the sibling’s death in an ICU or ER or at home shortly after withdrawal of life-prolonging technology. Children completed the Child Depression Inventory and Spence Children’s Anxiety Scale. Sibling cause of death was collected from hospital records.

**Results:**

At 2 and 4 months, 45% to 54% of mothers’ and 53% to 84% of fathers’ ratings of their child’s health “now” were higher than their children’s ratings. Child health ratings were lower for: children with greater depression; fathers whose children reported greater anxiety; mothers whose child died of a chronic condition. Children’s ratings of their health “now vs before” their sibling’s death did not differ significantly from mothers’ or fathers’ ratings at 2 or 4 months. Black fathers were more likely to rate the child’s health better “now vs before” the death; there were no significant differences by child gender and cause of death in child’s health “now vs before” the death.

**Conclusions:**

Children’s responses to a sibling’s death may not be visually apparent or become known by asking parents. Parents often perceive their children as healthier than children perceive themselves at 2 and 4 months after sibling death, so talking with children separately is important. Children’s perceptions of their health may be influenced by depression, fathers’ perceptions by children’s anxiety, and mother’s perceptions by the cause of sibling death.

## Introduction

Death of a child is an extremely painful experience for parents and siblings and has lasting effects on the family for years [1,2]. In the United States, more than 42,000children (newborn-19 years) die each year [3], half of these deaths occurring in hospitals [3] and most in intensive care units [4]. Each year approximately 2 million children experience the loss of a sibling, leaving 25% in need of clinical intervention and more than 50% with significant behavioral problems [5,6]. However the research on children’s responses to the death of a sibling is limited, heavily focused on sibling deaths from cancer and reported from the parent’s perspective.

The primary aim of this study in children whose sibling died in a neonatal intensive care unit (NICU), pediatric intensive care unit (PICU), Emergency Room (ER) or at home shortly after withdrawal of life-prolonging technology was to compare parents’ and children’s perceptions of the surviving child’s health at 2 and 4 months post sibling death. A secondary aim was to identify child factors (age, gender, race/ethnicity, anxiety, depression) and the sibling’s cause of death related to these perceptions.

### Children’s physical and emotional responses

Most physical and behavioral changes, morbidity and mortality in surviving children occur within the first year after the sibling’s death [7] but effects may remain for decades [8]. Children report experiencing anxiety, depression, guilt, and changes in school work, activities/interests, personality, goals and life perspectives during the first year after the sibling’s death [9]. Changed behaviors include acting out, aggression, withdrawal, lower school functioning and changed relationships with peers, parents and their other siblings [10–12]. Some children attempt suicide [10,11]. Children also experience allergies, asthma, somatic behavior including stomach-aches and headaches [13], daytime enuresis, nausea, and skin rashes [10]. These changes may be affected by the age and gender of the surviving child, the closeness of relationships with the deceased, the nature of the death and the child’s support systems. Children may experience negative effects of their sibling’s death at the time and again as adults [8].

### Parents’ vs children’s perceptions

The research on children’s responses to sibling death is largely from the parent’s perspective [11]. Some studies of sibling deaths from cancer have shown that parents’ and children’s perceptions of the child’s health differ, with parents reporting more negative changes in their child’s physical and emotional behavior than the child reported [9]. Alternately, children reported more positive changes that led to personal growth and more changes in sibling and peer relationships than parents reported [9].

### Age, gender, race, cause of death

Children’s understanding of death varies with their developmental level. Young children do not understand the permanence of death and may expect the dead sibling to come alive [14]. Children's understanding of death matures considerably by the time they reach adolescence which may affect the signs and symptoms they manifest. Adolescents reported higher self-injury behaviors and higher rates of suicide during the first year after death of a loved one [15,16], and grief and behavior changes up to 2 years after a violent or sudden sibling death [17].

Girls with a sibling loss reportedly exhibited greater anxiety, depression, withdrawn behavior and attention and thought problems than boys [18]. Worden, Davies, and McCown [19] found boys to be at higher risk for emotional and behavioral problems after a parent’s death than after a sibling’s death. However, Lehman et al. [20] found that children regardless of gender were more negatively affected by a sibling’s death than a parent’s death.

Very few studies explore parent and child ratings of children’s health after a sibling’s death across racial groups. Existing studies have large samples of White parents and children. One study [21] examined Caucasian and Black African American bereaved college students’ grief. Black students maintained a stronger continuing bond with the deceased, had more complicated grief symptoms and spent less time speaking to others or getting professional support than Caucasian students.

Cause of death (acute or chronic) may affect children’s physical and emotional response to sibling loss [22,23].After sibling loss due to a sudden illness such as Sudden Infant Death Syndrome, 54% of children exhibited physical and emotional symptoms for more than a year [24]. In addition, death in ICU and emergency room environments easily add to the stress of parents and siblings who may see the dying child in pain, frightened, and being cared for by new and unfamiliar people, unfamiliar machines and tubes, and high noise levels [25].

In summary, death of a sibling has short-lived and long-term physical and emotional effects on surviving children’s health. Understanding surviving children’s health and what factors may affect it is important for treatment and clinical care. However, the limited research in this area is from parent report, lacks the children’s perspective, is heavily focused on sibling cancer deaths and consists largely of White samples. The present study addresses these gaps by comparing parents’ and children’s reports of children’s health after a sibling’s death and testing the influence of children’s age, gender, race, anxiety and depression and the sibling’s cause of death on parents’ and children’s perceptions of the surviving children’s health.

## Materials and Methods

### Sample

Surviving children and their parents were recruited from the NICUs, PICUs, and ERs of 4 South Florida children’s hospitals and newspaper obituaries throughout Florida. Inclusion criteria for the surviving children were: 1) 6 to 18 years old, 2) lived with the deceased sibling before PICU or ER admission or the mother of the deceased infant before NICU admission, 3) lived with the same parent(s) since the death, and 4) in their age-appropriate grade in school +/− 1 year. Both parent consent and child assent were necessary for the children to participate. English- and Spanish-speaking parents were at least 18 years old.

*Exclusion criteria* for the surviving child were: 1) conditions that rendered the child unable to participate verbally – e.g., severe brain damage, severe autism – but the family’s other children remained eligible, 2) living in foster care before or after the sibling’s death, and 3) death of a parent or more than one sibling in the same event because the children would be dealing with the death of more than one nuclear family member at the same time. In single-parent families, noncustodial parents were invited to participate if the custodial parent provided his/her name and contact information.

### Procedure

Following University and clinical site IRB approvals, clinical consultants weekly identified families of children (newborn to 18 years) who died in their hospital’s NICU, PICU, or ER or who were discharged home after withdrawal of life-prolonging technology, and who had 1 or more surviving children 6 to18 years old. A letter explaining the study in Spanish and/or English was sent to the family identifying the study’s research assistants (RAs) and providing the study’s office phone numbers and email address. About 1 week after the letter was sent, an RA called the family, screened for eligibility, and determined family willingness to participate. At the first data collection visit (2 months after the death) to the family’s home, the RA again explained the study to parent(s) and eligible children, answered their questions and obtained signed consent from parents and assent from children for participation.

Families of deceased children were also recruited from online obituary notices in Florida. The RAs searched for the family’s name in online databases (e.g. White Pages, InteliusTM), then worked to narrow the list of families with the same name. RAs searched for the family and/or deceased child on GoogleTM, FacebookTM and similar websites to pinpoint the correct family (or a family relative); obtain more reliable addresses, phone numbers and emails; and identify families not eligible for the study. Families who appeared to meet study eligibility were sent a letter about the study. The process described above for clinical site enrollment was then followed. All families were given a booklet of resources for grieving children, parents and grandparents.

At 2 (T1) and 4 (T2) months post sibling death, RAs read the children’s items to them in English to minimize the time for each child interview and the effect of the child’s reading ability/level on their responses. Parent questionnaires were administered in the language chosen by the parent (English or Spanish), including the family demographic form. Spanish-speaking parents were interviewed by an RA fluent in both English and Spanish. The research team (RAs, co-investigators) was trained by the principal investigators (PIs) and a child clinical psychologist in the study protocol, use of study instruments, how to recognize distress in parents and children, how to respond and any reporting procedures that might be needed.

### Measures

Parent and surviving child demographic data included age, gender, education, and self-identified race/ethnicity (“White non-Hispanic,” “Black non-Hispanic,” or “Hispanic/Latino”); parent’s marital status and family annual income. The sibling’s cause of death was obtained from the deceased’s hospital record. Surviving children and their parents rated the surviving child’s health. Children also rated their symptoms of anxiety and depression.

Child Health was measured using a 2-item Health Now scale and 1 item comparing health now to health before the sibling’s death at 2 and 4 months after sibling death. The Health Now scale has 2 items: perception of the child’s health “now” and “now in comparison to other children their age.” At both 2 and 4 months post-death the child and parents separately rated these two items from 1 “poor” to 10 “excellent”. Higher scores on the summative Health Now scale indicate better perceived health. Internal consistency reliabilities at T1 and T2 were .69 and .65 for children, .91 and .83 for mothers, and .97 and .83 for fathers, respectively.

Parents and children also rated the child’s health “now compared to before the sibling’s death” on a scale from 1 “much worse than before [the death]” to 5 “about the same” to 10 “much better than before [the death].” These ratings were categorized as “worse than before” (1–3), “same as before” (4–6), and “better than before” (7–10).

Child Anxiety. The Spence Children’s Anxiety Scale (SCAS) [26] measures the intensity of the child’s anxiety symptoms in the previous 2 weeks. Children rated the SCAS’s 45 items from 0 “Never” to 3 “Always.” The SCAS takes about 15 minutes to complete. Spence reports a coefficient alpha of .93 for the total scale. In this study, coefficient alphas for the total scale were .90 and .91 at 2 and 4 months, respectively. Spence [26] recommends cut values (normal, elevated) based on the child’s gender and age.

Child Depression The Child Depression Inventory (CDI) [27] was used to measure the intensity of the child’s depressive symptoms in the previous 2 weeks. The CDI has 27 items written at a 1st grade reading level that children (6 – 17 years old) rate from 1 – 3. It takes 5–10 minutes to complete. In a large ethnically diverse normative sample, Kovacs [27] reports internal consistencies of .71 to .89 and test-retest correlations of .74 to .83 after a 2- to 3-week interval. In this study, coefficient alphas at 2 and 4 months were .72 and .77, respectively. Kovacs [27] recommends clinical cut values as 0 to 19 for “no depression,” 20–35 as “some depression,” and ≥ 36 as “severe depression.”

## Results

Sample. Sixty four surviving children (25 boys and 39 girls) who lost a brother or sister, and their 48 parents (35 mothers and 13 fathers) participated. See Table 1. Surviving children’s ages ranged from 6 to 17 years with a mean of 7.5 years (SD =3.26). Most were 6 to 9 years. Children’s ages were categorized as “6 to 9,” “10 to 12,” and “13 to 18” for some of the data analyses.

Parents’ ages ranged from 24 to 52 for mothers and 29 to 50 for fathers. Most parents were Black non-Hispanic, had some education beyond high school, and were married or partnered, ranging from 3 to 27 years. Almost half of the families reported an annual family income of less than $25,000. Most parents chose to complete the data collection forms in English (Table 1).

Ages of the 36 deceased siblings ranged from birth to 18 years (M = 7.9 years, SD = 6.44). Twenty two died in the PICU, 7 in NICU, 6 in ER and one died at home. Causes of death included congenital anomalies (33%), respiratory diseases (25%), neoplasms (17%), prematurity complicated by a superimposed illness (11%), motor vehicle crashes(6%), head trauma(5%) and drowning(3%). More than half of the sibling deaths (56%) were related to a chronic condition including brain tumor, leukemia, cystic fibrosis and systemic lupus erythematosus. Acute sibling deaths included motor vehicle or Jet Ski crash, head trauma with fractured skull, and encephalitis.

Health Now. Children often perceived their health to be poorer than their parents did. At two months, 45% of mothers’ and 84% of fathers’ ratings of their child’s Health Now were higher than their children’s self-ratings. At 4 months, 54% of mothers’ and 53% of fathers’ ratings were higher than their children’s self-ratings. Children’s Health Now ratings were compared to their mothers’ and fathers’ ratings with paired t-tests at 2 and 4 months (Table 2). Children rated their Health Now significantly lower than their fathers did at two months after the sibling’s death, but not at four months. Comparisons of children’s self-rated Health Now with their mothers’ ratings were not statistically significant at either time point.

At 2 months after the sibling’s death, 34% of the children had elevated anxiety, while at 4 months, 24% of the children did. At 2 months none of the children had depression in the clinical range, but 3.5% did at 4 months. Relationships of the children’s, mothers’ and fathers’ ratings of the child’s Health Now with the child’s age, gender, depression and anxiety and the sibling’s cause of death were tested with Pearson correlations (Table 3). At 2 months, children’s lower ratings of their Health Now were related to greater child-reported depression. Fathers’ lower ratings were related to greater child-reported anxiety. Mothers’ ratings were lower if the sibling died from a chronic condition. Other correlations at 2 months and all correlations at 4 months were not statistically significant.

Relationship between Health Now ratings and the child’s race/ethnicity was tested with one way ANOVA. At 2 months, fathers’ ratings of the child’s Health Now were significantly different by the child’s race/ethnicity, F_2,16_ = 4.31, p = .03. However, post hoc comparisons with the Tamhane test for unequal variances failed to identify which racial/ethnic groups were significantly different. None of the other Health Now ratings were significantly different by racial/ethnic group.

Health Now vs Before. Children’s categorized ratings (worse, same, better) of their health now vs before their sibling’s death did not differ significantly from their mothers’ or fathers’ categorized ratings at 2 and 4 months with X^2^ tests (Table 4). Post hoc effect sizes for these 4 tests ranged from 1.40 to 1.83, all with power > .99 [29]. In the mother-child pairs, 38 children and 42 mothers at 2 months and 49 children and 48 mothers at 4 months rated the child’s health as the same or better now than before the death. In the father-child pairs, 16 children and 17 fathers at 2 months and all of the children and their fathers at 4 months perceived the child’s health to be the same or better now than before the death.

Categorized child, mother, and father ratings of the child’s health now vs before the death (worse, same, better)at 2 and 4 months were compared by child age group, gender, racial/ethnic group, and sibling’s cause of death with X^2^ tests. Two were statistically significant. At 4 months, three of the 10- to 12-year-olds but none of the younger children (ages 6–9 years) and adolescents (ages 13–18 years) perceived their health now as poorer than before the sibling’s death(Table 4).Also at 4 months, Black fathers were more likely to rate the child’s health now as better than before the death, while White fathers rated the child’s health now as the same as before the death, X^2^ = 10.31, 2 df, p = .006.

Relationships of the children’s, mothers’ and fathers’ ratings of their child’s health now vs before the sibling’s death with child-reported depression and anxiety were tested with Pearson correlations. At 2 months, children’s lower ratings of their health now vs before the sibling’s death were related to greater child-reported depression at 2 months, r = −.35, p = .03. None of the other correlations at 2 and 4 months were statistically significant.

## Discussion

### Sample

The body of existing research in this area has focused on children’s responses to a parent’s death or a sibling’s death from cancer. This is one of the first studies to focus on children’s perceptions of their health following the ICU/ER death of their brother or sister and to compare children’s and parents’ perceptions of the surviving child’s health, especially in non-cancer deaths. In cancer deaths, parents and siblings may have time to say good-by, do things with the child before the death, have time to prepare for the pending loss, and often in comfortable surroundings. In ICU and ER deaths the child may be in pain, be attached to machines making communication difficult or impossible, there is often little time to say good-by, the environments have high light and noise levels, and unfamiliar staff adding to parents and siblings stress. In addition, other studies have compared parent and child self-report of the child’s physical and emotional health but not after a sibling’s death. This study also included all children in the family from 3 racial/ethnic groups willing to participate, while most studies include one child in a family with largely White and English-speaking samples.

Data demonstrated that parents often perceived their children as healthier than the children did. This was true for both Health Now and compared to before the sibling’s death, and at 2 and 4 months after the death, although few were statistically significant.

There are several possible reasons for these differences between parents’ and children’s perceptions of their child’s health. Parents may be overwhelmed with their own grief, making it difficult for them to recognize changes in their surviving children’s health. Parents may need to see their surviving children as healthy so they can continue to function. Parents often do not expect a strong reaction from their children, especially when the deceased sibling was a newborn and had never been home [14], so they may discount children’s reactions to the contrary. In addition, children are aware of parents’ grief and distress about the death and try to shield or protect their parents from further distress by not sharing how they feel [14]. Study findings of children perceiving themselves as in poorer health than reported by parents is consistent with early research on children’s responses after a parent’s death by Craft and Craft [30] and Sweeting and West [31]. However study findings are contrary to those reported by Foster and colleagues [9] who found parents reported more negative changes in children’s physical and emotional health than children reported.

Factors influencing ratings of children’s health now at 2 months after the sibling death varied by respondent. Child health now ratings were lower for children with greater depression, for fathers of children who reported greater anxiety, and for mothers whose child died due to a chronic condition. Mothers may not be able to focus on their other child (ren) during the sibling’s treatment for a chronic condition and subsequent death. Fathers’ and children’s ratings appear to be based more on the child’s mental health than mothers’ ratings. Children’s lower ratings of their health now vs before the sibling’s death were related to greater child-reported depression at 2 months and age at 4 months. Some of the 10- to 12-year-olds reported their health now to be poorer than after the sibling’s death, but all of the 6- to 9-year-olds and 13- to 18-year-olds perceived their health now as the same or better than before their sibling’s death. Children’s self-rated health now vs before the death did not differ significantly by gender or their sibling’s cause of death.

Black fathers were more likely to rate the child’s health now as better than before the death, while White fathers rated the child’s health as the same as before the death. It is well documented that in the African American culture death is not viewed as final but as part of life’s continuum [21]. Maintaining a continuing connection with the deceased child may have had an influence on Black fathers’ perceptions of their surviving child’s health. In addition, as Laurie and Neimeyer [21] found in their study of African American and White college students, African American students had strong and supportive kinships following the death of loved ones. Such support may influence perceptions of one’s health after tragedy.

### Limitations

This study was conducted with 3 racial/ethnic groups, Black non- Hispanic, White non-Hispanic and Hispanic, however 50% of the sample was Black non-Hispanic. It is not clear if the study results would hold in samples with different racial/ethnic makeup. Data on surviving children’s health are reports from children and parents. Future research including objective child health data such as visits to health care providers and health care costs would provide more insight into children’s health after a sibling’s death. The limited number of fathers (13) in the study, while common in this type of research, may also be a limitation in describing their perceptions of their surviving child(ren)’s health. Study data were collected at 2 and 4 months after the sibling’s death; data collected at a time further from the death are needed.

### Practice Implications

Children’s responses to a sibling’s death may not be visually apparent or become known by asking parents. Many children have difficulty believing their brother or sister really died [14]. Some change their behaviors and their friends to escape the reality of the sibling’s death [32]. These changes may be most dramatic in the first year after the death.

Elevated anxiety was present in 34% of study children at 2 months and 24% at 4 months after the sibling’s death. Only a few children in this sample experienced depressive symptoms severe enough to indicate clinical depression. However, these depressive symptoms negatively affected children’s perceptions of their health and may affect their ability to function successfully in school, at home, and on the job. Periodic outreach to children after death of their brother or sister and their parents, especially in the first year, may help to uncover children’s physical and mental health needs.

Children need someone to understand them and to listen to what has happened to them and not judge them or their behavior. Asking children how they are doing when they are not with their parents is important since children may not want to admit their feelings in front of parents in an effort to protect parents from any further distress associated with losing a child. Asking children about their health, somatic complaints including headaches and stomach aches, anxiety, depression or sadness and about their school progress, their friends and any changes in schools, friends, or activities may provide important information on their progress. Girls may experience higher anxiety, depression, and withdrawn behavior while boys may be at higher risk for emotional and behavioral problems. Encouraging parents to share that their child has died with school nurses and/or guidance counselors is important since these professionals may help the surviving siblings in school with teachers and friends.

When discussing with the parents how the child is doing, study data indicate that mothers may provide an evaluation that is closer to the child’s perceptions than fathers. This is reasonable since mothers may spend more time with children providing greater opportunity to observe the child’s behavior and moods. Father seem to perceive a change when the child becomes anxious perhaps by displaying more activity and attention seeking behaviors. Resources that may help include support groups or camps for children who have lost a sibling where children can express their feelings with other children bereft of a sibling. Providing parents with contact information and websites of groups that focus on bereft children may be helpful to both children and families. Support groups for the parents can also be of great help.

## Conclusion

Death of a sibling has short-lived and long-term physical and emotional effects on surviving children’s health. Understanding surviving children’s health and what factors may affect it is important for treatment and clinical care. However, the limited research in this area is from parent report, lacks the children’s perspective, is heavily focused on sibling cancer deaths and consists largely of White samples. This study compared children’s and their parents’ perceptions of children’s health 2 and 4 months after sibling death and child factors of age, gender, race/ethnicity, anxiety, depression and sibling’s cause of death related to these perceptions. Data demonstrated that parents often perceived their children as healthier than the children did. At 2 and 4 months, percentages of mothers’ (45%, 54%) and fathers’ (84%, 53%) ratings of their child’s health were higher than their children’s ratings. Child health ratings were lower for: children with greater depression; fathers whose children reported greater anxiety; mothers whose child died of a chronic condition. Children’s ratings of their health now vs before their sibling’s death did not differ significantly from mothers’ or fathers’ ratings at 2 or 4 months. Black fathers were more likely to rate the child’s health as better now vs before the death; there were no significant differences by gender and cause of death in child’s health now vs before the death. While this study was conducted with Black non-Hispanic, White non-Hispanic and Hispanic parents and children, 50% of the sample was Black non-Hispanic. It is not clear if the study results would hold in samples with different racial/ ethnic makeup.

## Figures and Tables

**Table 1:** Parent, child, and family characteristics

Parents		Mothers (n = 35)	Fathers (n = 13)
Age [M (SD)]		36.8(7.33)	40.6 (6.31)
Race/Ethnicity [n (%)]	Black non-Hispanic	15(43%)	6(46%)
	White non-Hispanic	9(26%)	4(31%)
	Hispanic	11(31%)	2(15%)
	Mestizo	0 (0%)	1(8%)
Education [n (%)]	High School or Less	11(31%)	4(31%)
	Vocational or Some	11(31%)	5(38%)
	College		
	College Graduate	13(38%)	4(31%)
Primary Language [n (%)]	English	32(91%)	12(92%)
	Spanish	3(9%)	1(8%)
Relationship Status [n (%)]	Married/partnered	30 (86%)	12 (92%)
	Single or widowed	5 (14%)	1 (8%)
		Families (N = 36)	
Annual Family Income [n (%)]	Less than $25,000	16 (44%)	
	$25,000 to $59,999	11 (31%)	
	$60,000 to $99,999	2 (6%)	
	$100,000 and above	5 (13%)	
	Not reported	2 (6%)	
Insurance Type [n (%)]	Public	18(50%)	
	Private	7(19%)	
	Self-Pay	2(6%)	
	Notreported	9 (25%)	
Cause of Sibling Death [n (%)]	Acute	16 (44%)	
	Chronic	20 (56%)	
		Male (n= 25)	Female (n= 39)
Age [n (%)]	6–9 years	11 (44%)	16 (41%)
	10–12 years	6 (24%)	12 (31%)
	13–18 years	8 (32%)	11 (28%)
Race [n (%)]	White non-Hispanic	7 (28%)	11 (28%)
	Black non-Hispanic	13 (52%)	19 (49%)
	Hispanic	5 (20%)	9 (23%)

**Table 2:** Children’s, mothers’, and fathers’ ratings of child health now at two and four months.

Time Post-Death		Child	Mother		Post-Hoc	
	Health Now	M (SD)	M (SD)	Paired t	E.S.	Power
2 months	(42 pairs)	16.1 (3.53)	16.6 (3.89)	0.73	0.11	0.18
4 months	(48 pairs)	16.3 (3.34)	17.3 (3.42)	1.79	0.26	0.71
		Child *M (SD)*	Father *M (SD)*	*Paired t*	E.S.	Power
2 months	(19 pairs)	13.9 (3.62)	17.4 (3.10)	3.69*	0.85	0.99
4 months	(15 pairs)	16.1 (3.78)	17.3 (2.47)	1.79	0.46	0.69

* p = .002

**Table 3:** Factors related to ratings of child health now 2 and 4 months post sibling death.

Time Post Sibling Death	Child Health Now	Child age	Gender	Chronic vs Acute Cause of Death	Depression 2 months	Anxiety 2 months
2 months	Child Rating	−.01	.06	.09	−.42**	−.13
	Mother Rating	−.13	.12	.39**	−.29	−.20
	Father Rating	.41	−.18	.07	−,24	−58*
Time Post Sibling Death	Child Health Now	Child age	Gender	Chronic vs Acute Cause of Death	Depression 4 months	Anxiety 4 months
4 months	Child Rating	−.20	−.01	−.16	−.24	−.13
	Mother Rating	.01	−.08	.12	−.01	−.13
	Father Rating	.18	−.23	.26	.07	.14

* p< .05

** p < .01

**Table 4:** Ratings of health now vs before the death 2 and 4 months post sibling death.

Child Rating (2 months)	Mother Rating (2 months)				Father Rating (2 months)			
	Worse	Same	Better	X^2^	Worse	Same	Better	X^2^
Worse	0	4	0	0.84	0	1	0	0.71
Same	0	22	2		0	11	2	
Better	0	13	1		0	3	0	
Child Rating (4 months)	Mother Rating (4 months)				Father Rating (4 months)			
	Worse	Same	Better	X^2^	Worse	Same	Better	X^2^
Worse	0	2	0	7.03	0	0	0	3.07
Same	0	26	5		0	10	1	
Better	3	11	4		0	2	2	
